# Comparison of Solite RE Black and WaveOne Gold File Systems for Removal of Root Canal Filling Material: An Ex Vivo Nano CT Study

**DOI:** 10.3390/dj13120547

**Published:** 2025-11-21

**Authors:** Sruthi Sairaman, Pradeep Solete, Delphine Priscilla Antony Selvanayagam, Swathi Suresh, Surendar Ramamoorthi, Ahmed El-Kabbaney, Margherita Tumedei, Massimo Del Fabbro

**Affiliations:** 1 Department of Conservative Dentistry and Endodontics, Saveetha Dental College and Hospitals, Saveetha Institute of Medical and Technical Sciences, Saveetha University, Chennai 600077, India; sruthisairaman@gmail.com (S.S.); delphy.priscilla@gmail.com (D.P.A.S.); swathisureshkc18@gmail.com (S.S.); 2 Alrass General Hospital, Qassim Health Cluster, Ministry of Health, Alrass 58883, Saudi Arabia; drsurendar@gmail.com; 3 Department of Endodontics, Faculty of Dentistry, Mansoura University, Mansoura 35516, Egypt; ahmedkappany2001@mans.edu.eg; 4 Department of Biomedical, Surgical and Dental Sciences, University of Milan, 20122 Milan, Italy; margherita.tumedei@unimi.it; 5 Fondazione IRCCS Ca’ Granda Ospedale Maggiore Policlinico, 20122 Milan, Italy

**Keywords:** gutta-percha removal, nano-CT, remaining filling material, retreatment, Solite RE Black

## Abstract

**Background/Objectives:** Non-surgical retreatment aims to remove infected filling material, prepare the canal for disinfection, and re-obturate. This ex vivo study evaluated the residual filling material and dentin volume removed after retreatment using a rotary or reciprocating system. **Methods:** Twenty mesial roots of mandibular molars with two independent, straight canals were decoronated to a 16 mm working length and obturated. Pre-operative nano-CT scans measured the initial dentin and filling material volumes. Specimens were randomly assigned to two groups: Solite RE Black system (RE1-30/0.08; RE2-20/0.06) and WaveOne Gold—small file (20/0.07). Post-operative nano-CT scans were superimposed to calculate the remaining filling material and dentin volume removed. Data are presented as the mean ± SD, and groups were compared using independent *t*-tests. **Results:** Neither system completely removed the intracanal filling material. The mean residual filling material volume was 1.42 ± 0.21 mm^3^ (Solite RE Black) vs. 1.56 ± 0.27 mm^3^ (WaveOne Gold), representing approximately 14% vs. 16% of the initial filling material, respectively (*p* < 0.05). Mean dentin volume removed was 0.95 ± 0.18 mm^3^ vs. 1.12 ± 0.22 mm^3^, with no significant difference (*p* > 0.05). **Conclusions:** In single-rooted, straight canals, Solite RE Black removed more filling material than WaveOne Gold, while dentin loss was similar. These findings are specific to straight canals, and further studies are required for curved or multi-rooted teeth.

## 1. Introduction

Non-surgical root canal retreatment aims to remove existing gutta-percha and sealer from the root canal system to enable adequate disinfection through reinstrumentation and irrigation before re-obturation [1]. The amount of residual filling material after reinstrumentation varies widely, ranging from 4% to 45%, according to the literature [2,3,4]. These remnants can serve as a nidus for reinfection, contributing to endodontic failure. Numerous hand and rotary instrumentation techniques have been proposed to improve the efficiency of filling material removal; however, no single method has proven completely effective [5,6,7].

Previous studies comparing rotary and reciprocating systems for retreatment have yielded conflicting findings. Some investigations reported superior cleaning efficiency with reciprocating files due to their larger cutting angles and reduced torsional stress, while others demonstrated better filling material removal with continuous rotary systems because of their sustained rotational motion and greater canal wall contact [8,9,10]. The diversity in canal anatomy, file design, and sealer composition likely accounts for this variability. Endodontic treatment may cause considerable dentin loss across the tooth structures, making teeth more susceptible to vertical root fracture, which remains a significant clinical complication, either during or after root canal therapy [11,12,13,14,15]. Different file systems may variably preserve dentin structures. Therefore, further comparative analysis between contemporary rotary and reciprocating systems remains warranted.

The Solite RE Black system (Solite Dental, Choolaimedu, Chennai, India) is a recently developed two-file rotary retreatment system (RE1 and RE2) featuring heat-treated NiTi metallurgy, variable taper, and continuous rotation kinematics, designed to enhance flexibility and cleaning efficiency while preserving dentin. In contrast, the WaveOne Gold system (Dentsply Sirona, Charlotte, NC, USA) operates in a reciprocating motion and employs gold heat-treated NiTi alloy with a variable taper to improve cyclic fatigue resistance and reduce canal deformation. WaveOne Gold was selected as the comparator system due to its well-documented clinical use and proven retreatment performance in prior studies. Given these differing kinematic and metallurgical properties, the present ex vivo study aimed to evaluate the residual filling material and the volume of dentin removed after retreatment with the Solite RE Black system and WaveOne Gold reciprocating system using nano-computed tomography analysis. Nano-CT is a technological advancement of standard micro-computed tomography, featuring a markedly reduced focal spot size of 400 nm compared to the conventional 5–50 μm range in micro-CT [16]. 

The null hypothesis of this study is that the Solite RE Black system and the WaveOne Gold reciprocating system would exhibit comparable efficacy in the removal of previous filling material and similar amounts of dentin removal during non-surgical root canal retreatment as assessed by nano-CT analysis.

## 2. Materials and Methods

**Figure 1:**
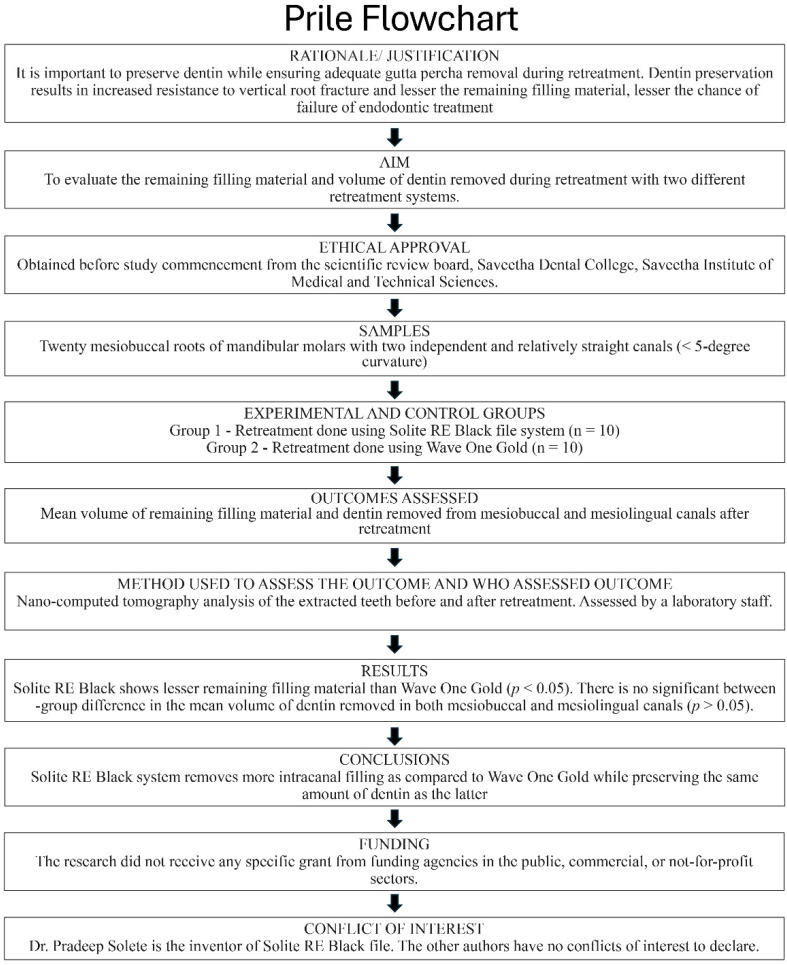
Methodology flowchart according to the PRILE guidelines.

This study was conducted following approval from the Scientific Review Board Committee (SRB/SDC/ENDO-2105/24/205, approval date: 10 July 2024) and the Institutional Human Ethical Committee (IHEC/SDC/ENDO-2105/24/162, approval date: 21 July 2024). The study was conducted in accordance with the Declaration of Helsinki for medical research involving human subjects (last update: October 2013). The present manuscript was written according to Preferred Reporting Items for Laboratory Studies in Endodontology (PRILE) 2021 guidelines, as detailed in the flowchart in Figure 1 [17]. 

### 2.1. Sample Selection

The sample size was determined based on a prior study that examined the remaining filling material between rotary and reciprocation systems using micro-CT analysis [18]. An a priori calculation indicated that 4 samples (teeth) per group would be necessary with an expected effect size of 1.2, standard deviation of 0.15 mm^3^, alpha = 0.05, and power = 0.95, using G*Power v3.1 software. However, the group size was increased to 10 teeth per group (20 mesiobuccal and 20 mesiolingual roots total) to allow for potential specimen loss or exclusion during preparation and imaging, to increase the precision and external validity of the results, and to ensure adequate power for secondary outcomes (such as dentin volume change) and for non-parametric or subgroup analyses. The present study therefore included twenty mesiobuccal roots and twenty mesiolingual roots from lower molars with relatively straight canals and two independent roots (curvature < 5°) [19]. Teeth with abnormal canal/root morphology, complex intricacies, prior root filling, or craze lines were excluded. Radiographic evaluations were performed on all teeth to identify and remove specimens with deformities. Diamond discs were used to standardize the working length to 16 mm after decoronation (Strauss & Co, Raanana, Israel). All extracted teeth used in this study were collected from patients undergoing routine dental extractions at university affiliated hospital, following written informed consent for the use of the teeth in research. Teeth were de-identified and stored in 0.1% thymol solution at 4 °C until use to prevent microbial growth.

### 2.2. Preparation of Root Canal

A single, well-trained operator performed all endodontic procedures to ensure consistency. Access cavities were prepared using an Endo Access Bur 2 (Dentsply Maillefer, Ballaigues, Switzerland), and canal patency was confirmed using size 10K stainless steel files (Mani, Utsunomiya, Japan), which were advanced until the tip was visible at the apical foramen. The working length was established 0.5 mm short of this point. Canal shaping was performed using the ProTaper Gold system (Dentsply Maillefer, Ballaigues, Switzerland) up to the F1 file. For Solite RE Black, RE1 was operated at 300 rpm, 2 N·cm torque, and RE2 at 350 rpm, 1.8 N·cm torque, in continuous rotation mode as per the manufacturer’s instructions. WaveOne Gold small file was used with the Dentsply Sirona endomotor in ‘WaveOne Gold’ reciprocation mode (170° CCW/50° CW). During instrumentation, canals were irrigated with a total of 20 mL of 3% sodium hypochlorite (NaOCl) (Prime Dental, Thane, India) per specimen, delivered at each file change. Upon completion of the chemomechanical preparation, a final irrigation protocol was followed: 5 mL of 17% ethylenediamine tetraacetic acid (EDTA) (Prime Dental, Thane, India) for 3 min, followed by 5 mL of 3% sodium hypochlorite (NaOCl) for 2 min, and finally 1 mL of distilled water. The canals were then dried using sterile paper points (META Biomed, Chungcheongbuk-do, Korea). Root canal obturation was performed using corresponding F1 gutta-percha (GP) cones (Dentsply Maillefer, Ballaigues, Switzerland) and AH Plus sealer (Dentsply DeTrey GmbH, Konstanz, Germany) utilizing the matched taper technique. Retreatment was performed using a standardized instrumentation technique, but retreatment time was not measured, as the study focused on the volumetric assessment of filling removal and dentin preservation. Excess gutta-percha was removed at the canal orifice using a heated plugger (No. 1 Glick blade plugger, HuFriedyGroup, Chicago, IL, USA). The quality of obturation was verified using periapical digital radiographs (buccolingual and mesiodistal views) (RVG 5200, Carestream Dental, Atlanta, GA, USA). Samples exhibiting voids or inadequate fillings were excluded from the study. To seal the access cavity, a composite material (Neo-spectra ST, Dentsply Sirona, Charlotte, NC, USA) was applied. The specimens were then stored in Eppendorf microcentrifuge tubes containing distilled water at 37 °C and 100% humidity for two weeks for the sealer to set completely. Following this period, samples were retrieved, and pre-operative nano-CT analysis was performed prior to gutta-percha removal.

### 2.3. Nano-CT Analysis 

High-resolution nano-CT imaging was performed using the ultraprecise Bruker SKYSCAN 2214 system (Bruker Micro-CT, Kontich, Belgium). Scanning parameters were set to an exposure time of 1100 ms, with a voltage of 100 kV, power output of 10 W, and a current of 100 µA. The flat-panel detector executed a 360° rotation with 0.3° angular steps, enabling comprehensive image acquisition. Image reconstruction was performed using NRecon software v1.6.9 (Bruker Micro-CT, Kontich, Belgium), which employs a modified Feldkamp cone-beam algorithm. Several post-processing corrections were applied to enhance image quality, including:

- Gaussian smoothing filter (kernel = 2),
- Beam hardening correction at 40%,
- Post-alignment correction of 0.50,
- Ring artifact correction set to 10.

The beam-hardening correction (40%) and Gaussian smoothing filter (kernel = 2) were applied to minimize artifacts while preserving structural details. Binary segmentation used a histogram-based global threshold, applied consistently across all specimens. To ensure reliability, five randomly selected scans were reprocessed, showing <2% variation in volumetric measurements. Additionally, intra-observer repeatability was assessed by re-analyzing 10 specimens after two weeks, yielding ICC > 0.90, confirming excellent measurement reproducibility.

The grayscale images were standardized to a fixed resolution of 1944 × 3072 pixels, with a voxel size of 11.9996 nm.

Further image processing and analysis were performed using CTAn software v1.14.0 (Bruker Micro-CT). The region of interest (ROI) was defined to differentiate between the root canal filling material and dentin through binary segmentation. A consistent binary threshold value, determined from the histogram of each specimen, was applied across all nano-CT datasets to ensure standardization. Quantitative volumetric analysis of the segmented regions was carried out using CTVol v2.2.1 (Bruker Micro-CT, Bruker Corp., Billerica, MA, USA).

To validate the accuracy and reproducibility of the Nano-CT data, system calibration was performed according to the manufacturer’s standard protocol, including geometric alignment, detector calibration, and beam hardening correction. All specimens were scanned using identical acquisition and reconstruction parameters to ensure consistency. Binary segmentation was performed using histogram-based global thresholding, and the same threshold range was uniformly applied across all specimens to distinguish dentin from filling material. To verify volumetric stability, five randomly selected datasets were reprocessed, revealing less than 2% variation in measured volumes, confirming the reproducibility and reliability of the segmentation process.

After the volume measurements were obtained, to confirm the uniform distribution of initial obturation volumes between the groups, an unpaired *t*-test was conducted using SPSS software version 23 (IBM Corp., SPSS Inc., Chicago, IL, USA) version 23. The statistical analysis confirmed that the groups were evenly distributed, with no significant differences in median canal volumes between group I (9.87 ± 0.13 mm^3^) and group II (10.24 ± 0.35 mm^3^) (*p* > 0.05). 

### 2.4. Reliability and Repeatability Assessment

To ensure the reliability and reproducibility of the volumetric measurements, intra-observer repeatability and threshold validation were performed. Ten randomly selected specimens (25% of the total sample) were re-analyzed after a two-week interval by the same examiner, who was blinded to the previous measurements. The degree of agreement between the two measurements was assessed using the intraclass correlation coefficient (ICC), which demFonstrated excellent reliability (ICC > 0.90).

To minimize operator bias during segmentation, a single calibrated examiner performed all binary thresholding and volumetric analyses. The segmentation threshold for distinguishing filling material and dentin was determined using histogram-based global thresholding, and the same threshold range was consistently applied across all scans to ensure standardization.

For repeatability verification, five datasets were reprocessed independently by the same operator, yielding volume differences below 2%, confirming the stability of the segmentation protocol.

### 2.5. Root Canal Retreatment Procedure

Samples were randomly divided into two groups of 10 teeth each. Group 1 received the Solite RE Black system and Group 2 received WaveOne Gold. 

The retreatment procedures were then carried out in group 1 using the Solite RE Black system (RE1—30/0.08, variable taper, RE2—20/0.06 variable taper) and in group 2 using the WaveOne Gold (small file—20/0.07, variable taper). The procedure was carried out according to the manufacturer’s instructions, with no solvent used at any stage. Irrigation was performed with 20 mL of 3% sodium hypochlorite. After the completion of gutta-percha removal, the canals were irrigated with 5 mL of 17% ethylenediaminetetraacetic acid (EDTA) (Prime Dental, India) solution for 3 min, 5 mL of 3% NaOCl solution for 2 min, and 1 mL of distilled water, and then dried with paper points (META BioMed, Chungcheongbuk-do, South Korea). 

When the files reached the desired working length, the dentinal walls were smooth, and no filling material could be seen under an optical microscope (OPMI PICO, Carl Zeiss, Oberkochen, Germany), The retreatment was considered complete. For nano-CT, using the same settings, a second scan of every specimen was carried out with the SKYSCAN2214 scanner. The volumetric analysis of the residual filling material and the volume of dentin removed was then performed by superimposing the pre-operative and post-operative nano-CT. All the measurements were taken and noted by a laboratory staff member who was blinded, in an Excel sheet. 

### 2.6. Statistical Analysis

Statistical analysis was performed using SPSS software version 23 (IBM Corp., Chicago, IL, USA). Each canal was treated as an independent experimental unit. Normality of the data for both outcome variables (remaining filling material and dentin removed) was assessed using the Shapiro–Wilk test, confirming that parametric testing was appropriate. Descriptive statistics, including means, standard deviations (SDs), sample size, and 95% confidence intervals (CIs), are reported for all groups. Group comparisons were performed using independent Student’s *t*-tests, with *p*-values < 0.05 considered statistically significant. In addition, Cohen’s d effect sizes were calculated to assess the magnitude of differences between groups. All analyses are presented with the means ± SD, 95% CIs, *p*-values, and effect sizes to ensure full transparency and reproducibility.

## 3. Results

**Figure 2:**
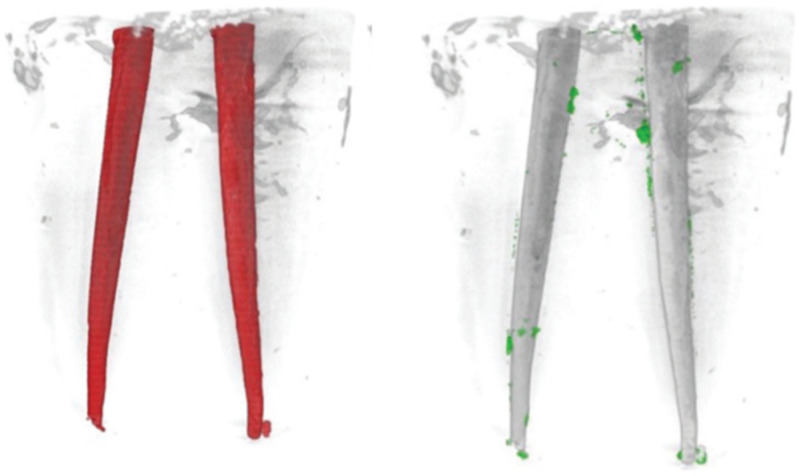
The merged pre-operative (**left**) and post-operative (**right**) nano-CT images of a sample from the Solite RE Black group are presented. In these images, the red areas (**left**) represent the obturated canal, the green areas (**right**) indicate the remaining filling material, while the gray areas represent the filling material that has been removed after retreatment.

**Figure 3:**
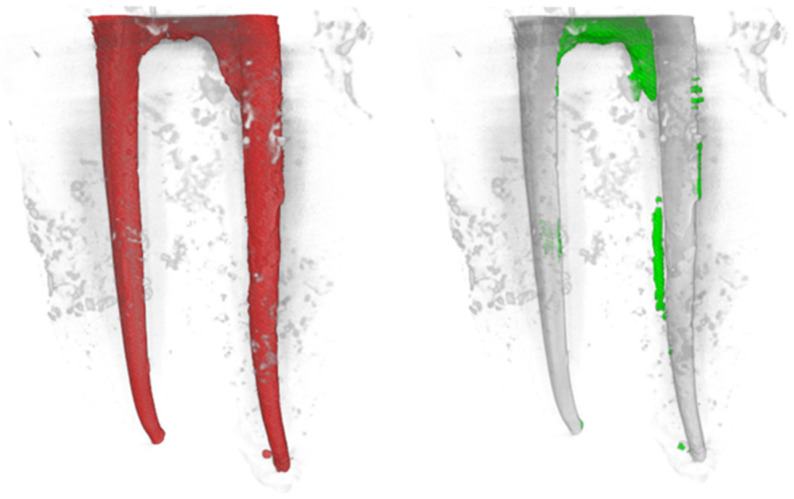
The merged pre-operative (**left**) and post-operative (**right**) nano-CT images of a sample from the WaveOne Gold group are presented. In these images, the red areas (**left**) represent the obturated canal, the green areas (**right**) indicate the remaining filling material, while the gray areas represent the filling material that has been removed after retreatment.

Nano-CT imaging revealed remnants of filling material in both the Solite RE Black (Figure 2) and WaveOne Gold (Figure 3) groups; neither of the file systems was able to completely remove the intracanal filling material.

The mean (±SD) volume of remaining filling material after retreatment was 1.42 ± 0.21 mm^3^ for the Solite RE Black system and 1.56 ± 0.27 mm^3^ for the WaveOne Gold system, showing a statistically significant difference (*p* < 0.05) (Table 1).

When the amount of dentin removed was analyzed, the mean (±SD) dentin loss was 0.95 ± 0.18 mm^3^ for Solite RE Black and 1.12 ± 0.22 mm^3^ for WaveOne Gold, with no statistically significant difference between groups (*p* > 0.05) (Table 2).

Box-and-whisker plots and standardized residual analysis were performed to assess intra-group variability and potential outliers. No extreme values (greater than ±3 SD) were detected, indicating homogeneity and consistency within each group.

## 4. Discussion

Endodontic retreatment shares the same objective as the primary treatment of infected root canals: to completely eradicate micro-organisms and achieve a three-dimensional seal using biocompatible materials. This process involves the removal of the existing root canal filling material, thorough disinfection of the root canal system, and the subsequent sealing of the canals [20]. The risk of failure between surgical and non-surgical endodontic re-treatment has no statistically significant difference according to the literature, thus making non-surgical endodontic retreatment the first-line treatment option in cases of failure of primary endodontic therapy because of its reduced invasiveness [21]. Nano-CT utilizes a high-power nanofocus X-ray source to characterize and quantify tissue microarchitectures precisely [22,23]. While micro-CT has traditionally been considered the regular modality for assessing endodontic procedures from canal morphology to obturation quality, recent research emphasizes the advanced capabilities of nano-CT [24]. This technology can detect more subtle changes than micro-CT [25]. 

Compared to the literature on using rotary systems for the removal of intracanal filling material during non-surgical retreatment, the data on using reciprocation files for the same are quite scarce [26,27,28]. Reciprocating files when used for retreatment have the advantage of using a lesser number of file sequences during treatment, thus being cost effective, as most rotary retreatment file systems require the further usage of additional finishing files before obturation, which is why the present study aimed to compare a relatively new rotary retreatment system to reciprocation files [26]. Earlier literature has reinstated that larger apical preparations can result in the better removal of infected dentin and deeper penetration of irrigants, which significantly reduces the microbiota from the canal system, thus ensuring good periapical healing [29,30,31].

Previous comparative studies have yielded conflicting findings regarding the efficacy of rotary versus reciprocating retreatment systems. Some authors have reported that reciprocating files, due to their alternating motion and enhanced flexibility, improve debris dislodgment and reduce procedural fatigue [32,33]. Others, however, have demonstrated that rotary systems achieve greater filling material removal due to continuous cutting engagement and higher torsional efficiency [34,35]. These inconsistencies underline that file design, alloy treatment, and motion dynamics collectively influence retreatment outcomes.

However, with the introduction of supplemental chemo-mechanical techniques like passive ultrasonic irrigation, it has been shown that there is also no difference in periapical healing with lesser preparation sizes, which preserves much more dentin compared to larger apical preparations [36]. To maximize the long-term survival of an endodontically treated tooth, the clinician must strive to preserve as much of the natural tooth structure as possible. Structural changes resulting from endodontic access and canal shaping, including altered cross-sectional geometry, increased canal curvature, and the loss of circumferential dentin, significantly affect the internal stress distribution within the root. These factors contribute to a heightened risk of fracture in endodontically treated teeth [37,38].

This study only used the small file from WaveOne Gold (WOG—20/0.07, variable taper) to standardize apical preparation sizes with the apical retreatment file from the Solite RE Black system (RE 2—20/0.06) and evaluate the cleaning efficiency in smaller apical preparations, thus preserving dentin. The selection of a retreatment system is critical in influencing the degree of dentinal loss, with larger preparations leading to greater root flexure and an increased risk of vertical root fractures [39]. 

The current study evaluated the dentin removed in limited samples in single rooted teeth with one canal, and the canal shaping was less subject to operator variability due to the relatively straight nature of the canals. Consistent with previous studies, our findings also indicate that neither of the file systems completely removed the intracanal filling material [18,40]. However, in the present study, significantly less remaining material was found with Solite RE Black compared to WaveOne Gold 20. Interestingly, in both groups, the mean volume of remaining filling material was slightly greater in the mesiobuccal canals compared to the mesiolingual ones. This could be attributed to the increased curvature in the former [41]. Nevertheless, despite the different taper size of the two types of files, there was no significant difference between groups in the mean volume of dentin removed. The evaluation of only two outcomes might represent a limitation of the present study. Assessment of other parameters, such as canal centering and apical transportation between the two systems, might yield further knowledge and provide better insight for the operator. The present study did not assess clinical performance parameters such as retreatment time, number of file passes, or instrument fatigue. These parameters, although clinically relevant, were beyond the scope of this nano-CT–based analysis, which focused on the quantitative evaluation of filling material removal and dentin preservation under standardized laboratory conditions. Future studies combining volumetric assessment with these practical efficiency measures would provide a more comprehensive understanding of the clinical performance of the retreatment systems.

Furthermore, the exclusion of additional procedural and performance-related parameters, such as canal centering ability, apical transportation, and debris extrusion, limits the comprehensiveness of the present findings. These parameters are crucial for assessing the shaping efficiency, safety, and biological implications of retreatment instruments. While nano-CT provides high-resolution volumetric data, the absence of a dynamic canal shaping evaluation restricts a full understanding of how each system behaves within complex canal morphologies. Future studies integrating nano-CT analysis with micro-computed or optical evaluation of canal transportation and apical extrusion would offer a more holistic understanding of file performance.

When compared with recent studies evaluating other reciprocating retreatment systems—such as Reciproc Blue, ProTaper Universal Retreatment, and XP-Endo Finisher R—our findings are partly consistent with reports that reciprocating files often leave residual filling material but can limit dentin removal due to controlled cutting efficiency [27,42]. Conversely, some studies have demonstrated superior filling removal by rotary systems, possibly due to their continuous motion and greater cutting contact area [27,43]. The present results therefore complement the existing literature by demonstrating that Solite RE Black achieved significantly less remaining material than WaveOne Gold under standardized conditions, supporting its effective retreatment potential while minimizing dentin loss.

The superior performance of the Solite RE Black system in removing residual filling material can likely be attributed to its continuous rotation motion, variable taper design, and cutting geometry, which allow for more effective engagement with the canal walls while minimizing unnecessary dentin removal. Its metallurgical properties may also contribute to flexibility and resistance to file fatigue, enhancing its efficacy in retreatment. Clinically, this indicates that Solite RE Black can achieve better cleaning efficiency without compromising dentin preservation, which is essential to reduce the risk of vertical root fractures and maintain long-term tooth integrity.

It must also be acknowledged that the instrument stress distribution, debris extrusion, and canal transportation may differ substantially, potentially influencing both clinical efficiency and safety outcomes. Further investigations on anatomically diverse specimens would be essential to validate the external applicability of the present findings.

It is important to note that one of the authors is listed as an inventor on a patent related to the Solite RE Black system. To ensure objectivity, all volumetric measurements were conducted by a blinded, calibrated examiner, with standardized binary thresholding applied uniformly across all scans. Intra-observer reliability was confirmed via reanalysis of 25% of the specimens (ICC > 0.90), and volumetric reprocessing demonstrated <2% variation, confirming the reproducibility and reliability of the measurements.

Throughout the retreatment procedures, no procedural errors such as ledge formation, canal transportation, or file separation were observed under microscopic supervision. However, as the study was conducted on standardized straight canals and focused primarily on volumetric assessment, these safety parameters were not quantitatively analyzed. Future studies involving canals of varied curvature and incorporating procedural deviation assessment would provide a more comprehensive evaluation of the safety and predictability of these systems. It is important to note that nano-CT imaging places a much greater demand on computer processing compared to micro-CT. Additionally, the study faced constraints in solely utilizing the nano-focus principle related to the size of the roots, adding an additional layer of complexity. Future studies should evaluate the performance of both rotary and reciprocating retreatment systems in anatomically diverse canals, including curved and multi-rooted teeth, to better simulate clinical conditions. Combining high-resolution volumetric analysis with assessments of canal transportation, apical extrusion, procedural deviations, and clinical efficiency parameters such as retreatment time and number of file passes would provide a more comprehensive understanding of retreatment system performance and safety.

## 5. Conclusions

In the present study of single-rooted, relatively straight canals, neither of the file systems completely removed the intracanal filling material, with Solite RE Black being more effective than WaveOne Gold. The volume of dentin removed by the two systems was equivalent. These findings are specific to straight canals, and further investigations are required to determine whether similar results apply to curved or multi-rooted teeth, where instrument performance and dentin removal may differ.

## Figures and Tables

**Table 1:** Volume of remaining filling material in both mesiobuccal and mesiolingual canals after retreatment in the Solite RE Black and WaveOne Gold groups. Data are expressed as the mean value and standard deviation; 95% confidence intervals and effect sizes are also reported.

Canal	Groups	Mean Volume of Remaining Filling Material, mm^3^	95% CIs, mm^3^	Cohen’s d	*p*-Value
Mesiobuccal	Solite RE Black	1.96 ± 0.48 (*n* = 10)	1.662, 2.258	2.15	0.03
	WaveOne Gold	4.37 ± 1.51 (*n* = 10)	3.434, 5.306		
Mesiolingual	Solite RE Black	1.46 ± 0.20 (*n* = 10)	1.336, 1.584	6.90	0.04
	WaveOne Gold	4.73 ± 0.64 (*n* = 10)	4.333, 5.127		

**Table 2:** Volume of dentin removed in both the mesiobuccal and mesiolingual canals after retreatment in the Solite RE Black and WaveOne Gold groups. Data are expressed as the mean value and standard deviation; 95% confidence intervals and effect sizes are also reported.

Canal	Groups	Mean Volume of Dentin Removed, mm^3^	95% CIs, mm^3^	Cohen’s d	*p*-Value
Mesiobuccal	Solite RE Black	0.708 ± 0.041 (*n* = 10)	0.683, 0.733	0.046	0.61
	WaveOne Gold	0.706 ± 0.046 (*n* = 10)	0.677, 0.735		
Mesiolingual	Solite RE Black	0.661 ± 0.043 (*n* = 10)	0.634, 0.688	0.408	0.73
	WaveOne Gold	0.645 ± 0.035 (*n* = 10)	0.623, 0.667		

## Data Availability

The data are available upon request from the corresponding author.
